# CTGF increases matrix metalloproteinases expression and subsequently promotes tumor metastasis in human osteosarcoma through down-regulating miR-519d

**DOI:** 10.18632/oncotarget.1998

**Published:** 2014-05-21

**Authors:** Hsiao-Chi Tsai, Hong-Lin Su, Chun-Yin Huang, Yi-Chin Fong, Chin-Jung Hsu, Chih-Hsin Tang

**Affiliations:** ^1^ Department of Life Sciences, National Chung Hsing University, Taichung, Taiwan; ^2^ Department of Orthopaedic Surgery, China Medical University Beigang Hospital, Yun-Lin County, Taiwan; ^3^ Graduate Institute of Clinical Medical Science, China Medical University, Taichung, Taiwan; ^4^ School of Chinese Medicine, College of Chinese Medicine, China Medical University, Taichung, Taiwan; ^5^ Department of Orthopedic Surgery, China Medical University Hospital, Taichung, Taiwan; ^6^ Graduate Institute of Basic Medical Science, China Medical University, Taichung, Taiwan; ^7^ Department of Pharmacology, School of Medicine, China Medical University, Taichung, Taiwan; ^8^ Department of Biotechnology, College of Health Science, Asia University, Taichung, Taiwan

**Keywords:** Osteosarcoma, MicroRNA, MMP, CTGF, Metastasis

## Abstract

Osteosarcoma, the most common primary malignant bone tumor, shows potent capacity for local invasion and distant metastasis. Connective tissue growth factor (CTGF/CCN2), a secreted protein, binds to integrins, modulates invasive behavior of certain human cancer cells. Effect of CTGF in metastasis of human osteosarcoma is unknown. We found overexpression of CTGF increasing matrix metalloproteinases (MMPs)-2 and MMP-3 expression as well as promoting cell migration. MicroRNA (miRNA) analysis of CTGF-overexpressed osteosarcoma versus control cells probed mechanisms of CTGF-mediated promotion of migration. Among miRNAs regulated by CTGF, miR-519d was most downregulated after CTGF treatment. Co-transfection with miR-519d mimic reversed CTGF-mediated MMPs expression and cell migration. Also, MEK and ERK inhibitors or mutants reduced CTGF-increased cell migration and miR-519d suppression. By contrast, knockdown of CTGF diminished lung metastasis *in vivo*. Clinical samples indicate CTGF expression as linked with clinical stage and tumor metastasis. Taken together, data show CTGF elevating MMPs expression and subsequently promoting tumor metastasis in human osteosarcoma, down-regulating miR-519d via MEK and ERK pathways, making CTGF a new molecular therapeutic target in osteosarcoma metastasis.

## INTRODUCTION

Osteosarcoma, the most common primary malignant bone tumor, ranks as main cause of cancer-related death in children and adolescents. It frequently localizes to the distal femur and proximal tibia [1]. Decades ago, with surgery as the sole treatment, diagnosis of osteosarcoma often portended death. Patients had only a 15-20% chance of cure [2]. In the 1980s and 1990s, chemotherapy remarkably augmented survival rates; patients today have about 65-70% chance of 5-year relapse-free survival, if the disease is localized [3]. Yet other patients will relapse (with pulmonary metastasis) within five years and have undetectable metastatic disease at diagnosis. Pulmonary metastasis is the chief cause of death from osteosarcoma [4]. Patients with metastases have poor prognosis: long-term survival rate of 10-30% [5]. How to reduce metastasis in osteosarcoma is essential to osteosarcoma therapy. Thus, it is necessary to develop novel approaches for treating metastasis, meaning a need to clarify the molecular mechanisms underlying pathogenesis and progression.

Tumor metastasis is a multistep process. During metastasis, tumor cells can through secrete some proteins, growth factors and cytokines associated with the extracellular matrix (ECM) [6], some metastatic potential gene and protein can allow tumor cells to escape from the primary tumor, migrate, and invade surrounding tissues, enter the vasculature, circulate and reach secondary sites, extravasate, and finally establish metastatic foci [7, 8]. Connective tissue growth factor (CTGF/CCN2) is a member of the CCN family. CCN proteins modulate cell-ECM interactions and exhibit multi cellular functions such as regulation of cell division, apoptosis, adhesion, motility, and ion transport [9, 10]. CTGF is a secreted protein that acts as a multifunctional signaling modulator in various biologic or pathologic processes [11]. In cancer cell, many studies have report that CTGF enhanced tumor development and progression [12-14]. On the other hand, CTGF also promoted cancer cell migration and metastasis such as breast cancer [15], nasopharyngeal carcinoma [16], hepatocellular carcinoma [17], and melanoma [18], but negatively regulated in neuron-glioblastoma [19] and oral squamous cell carcinoma [20]. However, the role of CTGF in osteosarcoma metastasis has not yet been clarified.

One of the beginning step of metastasis is primary tumors invade locally through surrounding ECM or stromal cell layers. Matrix metalloproteinases (MMPs) are zinc-dependent endopeptidases and have been regarded as major critical molecules assisting tumor cells invasion [21]. Many studies linked MMPs with cancer invasion, migration or cancer cell-mediated tissue remodeling were the major positive correlations with MMP levels [8, 22]. MMPs have also been think one of promising therapeutic targets for osteosarcoma patients [23]. The function or expression of MMPs was regulated by many factors, including ECM molecules, growth factors, cytokines, and chemokines. Some of studies have demonstrated CCN proteins regulate MMPs protein expression [24, 25]. However, the role of CTGF in MMPs expression in human osteosarcoma is largely unknown.

MicroRNAs (miRNAs) are small noncoding RNAs that are 19 to 25 nucleotides in lengths, miRNAs regulate gene expression through binding to 3' untranslated regions (UTRs) of target mRNAs, either targeting the transcripts for degradation or blocking their translation [26]. There are more and more miRNAs have been involved in the regulation of cancer metastasis [27], and there have been considerable interest in the posttranscriptional regulation of MMPs via miRNAs [28]. Therefore, we hypothesized that miRNA may be involved in CTGF-mediated MMPs expression and tumor metastasis in human osteosarcoma. The results show that CTGF increases MMPs expression and subsequently promotes tumor metastasis in human osteosarcoma by down-regulating miR-519d through MEK and ERK pathway.

## RESULTS

### CTGF enhances migration in human osteosarcoma

**Figure 1:**
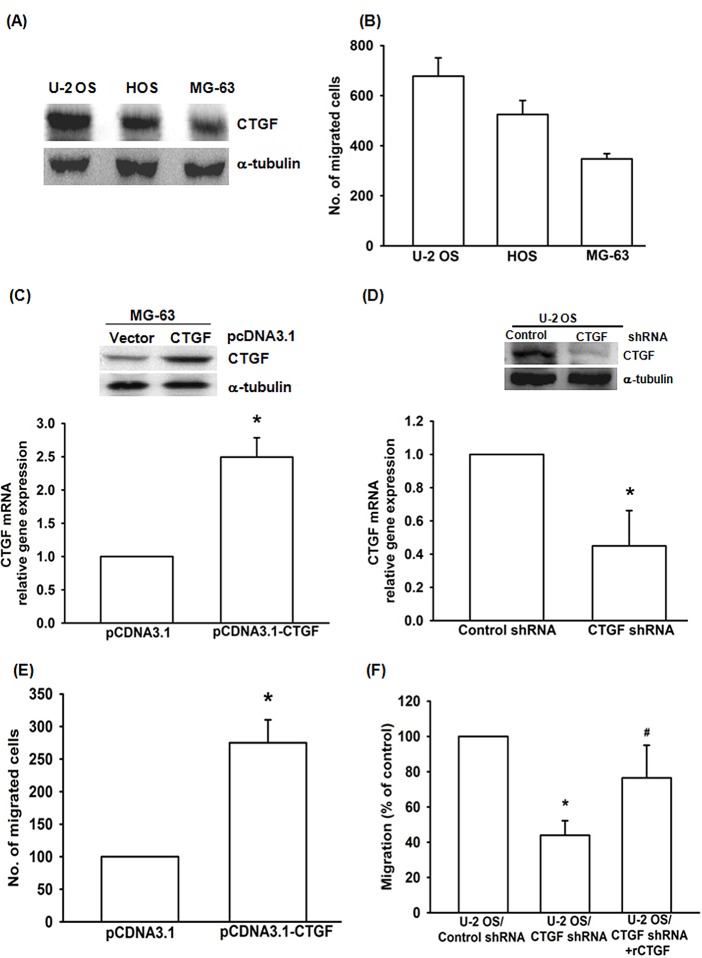
CTGF promotes migration in human osteosarcoma cells (A&B) CTGF expression and migratory activity in indicated cells was examined by western blot and Transwell assay. (C&E) CTGF-overexpressing MG-63 cells were established, using pCDNA3.1 vector. Protein and mRNA expression of CTGF and migratory activity were examined by western blot, q-PCR and Transwell assay. (D) Protein and mRNA expression of CTGF in indicated cells were examined by western blot and q-PCR. (F) U-2 OS/CTGF shRNA cells were incubated with vehicle or recombine protein of CTGF (rCTGF; 50 ng/ml), cell migration examined by Transwell assay, results expressed as mean ± SEM. *, p < 0.05 as compared with controls, and ^#^, p < 0.05 as compared with U-2 OS/CTGF shRNA group.

CTGF has been reported to promote cell migration and metastasis in variety of human cancer cells [29]. However, it is still not well-recognized whether CTGF stimulates tumor migration in osteosarcoma cells. First, we compared the protein expression of CTGF and migration ability in three osteosarcoma cell lines. Western blot revealed a higher level expression of CTGF in U-2 OS cells and lower level in MG-63 cells (Fig. 1A). U-2 OS cells were more migratory than MG-63 cells (Fig. 1B). Therefore, the migratory ability was positive corrected with CTGF expression in human osteosarcoma. To examine the link between CTGF expression and osteosarcoma migration, we established the CTGF-expressing plasmid (pcDNA3.1-CTGF). As shown in Fig. 1C and Supplementary Fig. S2B, the protein and mRNA expression were significant higher in CTGF-expressing cells (MG-63/CTGF and U-2 OS/CTGF) than in control cells (MG-63/vector and U-2 OS/vector). We also found the migration ability was dramatically increased in MG-63/CTGF cells (Fig. 1E). In contrast, shRNA-mediated knockdown of CTGF in U-2 OS/CTGF-shRNA cells and MG-63/CTGF-shRNA showed notably reduced CTGF expression, and the migration ability in U-2 OS cells (Fig. 1D&F; Supplementary Fig. S2A). On the other hand, incubation with recombinant human CTGF rescued CTGF-shRNA-reduced cell migration (Fig. 1F). These results indicate that CTGF promotes migration in human osteosarcoma cells.

### Repression of miR-519d by CTGF is critical in CTGF-mediated migration

**Figure 2:**
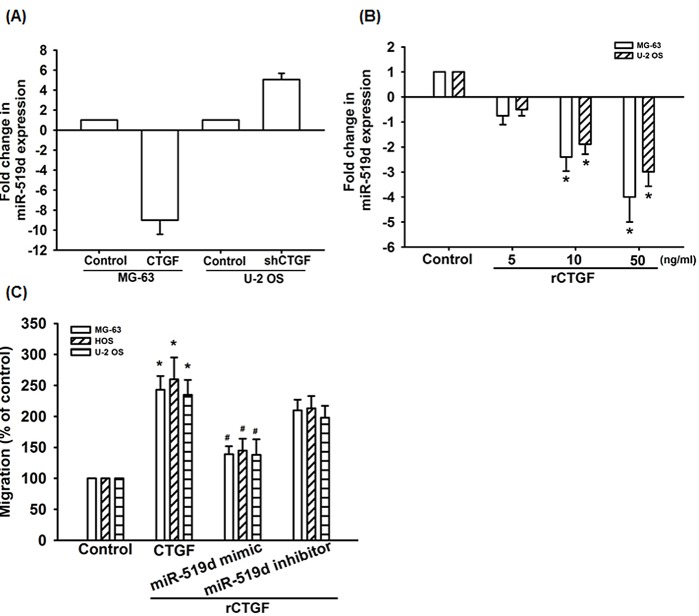
CTGF increases cell migration by suppressing miR-519d expression (A) The miR-519d expression in indicated cells was examined by qRT-PCR. (B) MG-63 cells and U-2 OS cells were incubated with rCTGF (5-50 ng/ml) for 24 h, miR-519d expression examined by qRT-PCR. (C) Cells were transfected with miR-519d mimic or inhibitor for 24 h and cell migration examined by Transwell assay. Results are expressed as mean ± SEM. *, p < 0.05 as compared with controls, and ^#^, p < 0.05 as compared with CTGF-treated control group.

MiRNA has been reported as an important regulator in cancer progression and metastasis [30]. In order to investigate miRNA differential expression in CTGF-overexpressed osteosarcoma cells, we used miRNome microRNA Profilers QuantiMir™ kit that contained 576 human miRNAs. Ten miRNAs were significantly up-regulated, and eight miRNAs were significantly down-regulated (Supplementary Table 1). Among the significantly regulated miRNAs, miR-519d was the most down-regulated in response to CTGF overexpression (Supplementary Table 1). We confirmed miR-519d expression using qRT-PCR, the miR-519d was down-regulated in MG-63/CTGF cells, and up-regulated in U-2 OS/CTGF-shRNA cells (Fig. 2A). In addition, directly applied CTGF in MG-63 cells and U-2 OS cells for 24 h reduced miR-519d expression in a concentration-dependent manner (Fig. 2B). To further verify the direct effect of miR-519d on cell motility, we transiently transfected the miR-519d mimic or inhibitor into osteosarcoma cells, and found miR-519d mimic but not inhibitor blocked the CTGF-induced cell migration (Fig. 2C). Taken together, CTGF increases osteosarcoma migration through down-regulating miR-519d.

### *MMP-2* and *MMP-3* are the downstream target genes in CTGF-regulated miR-519d

**Figure 3:**
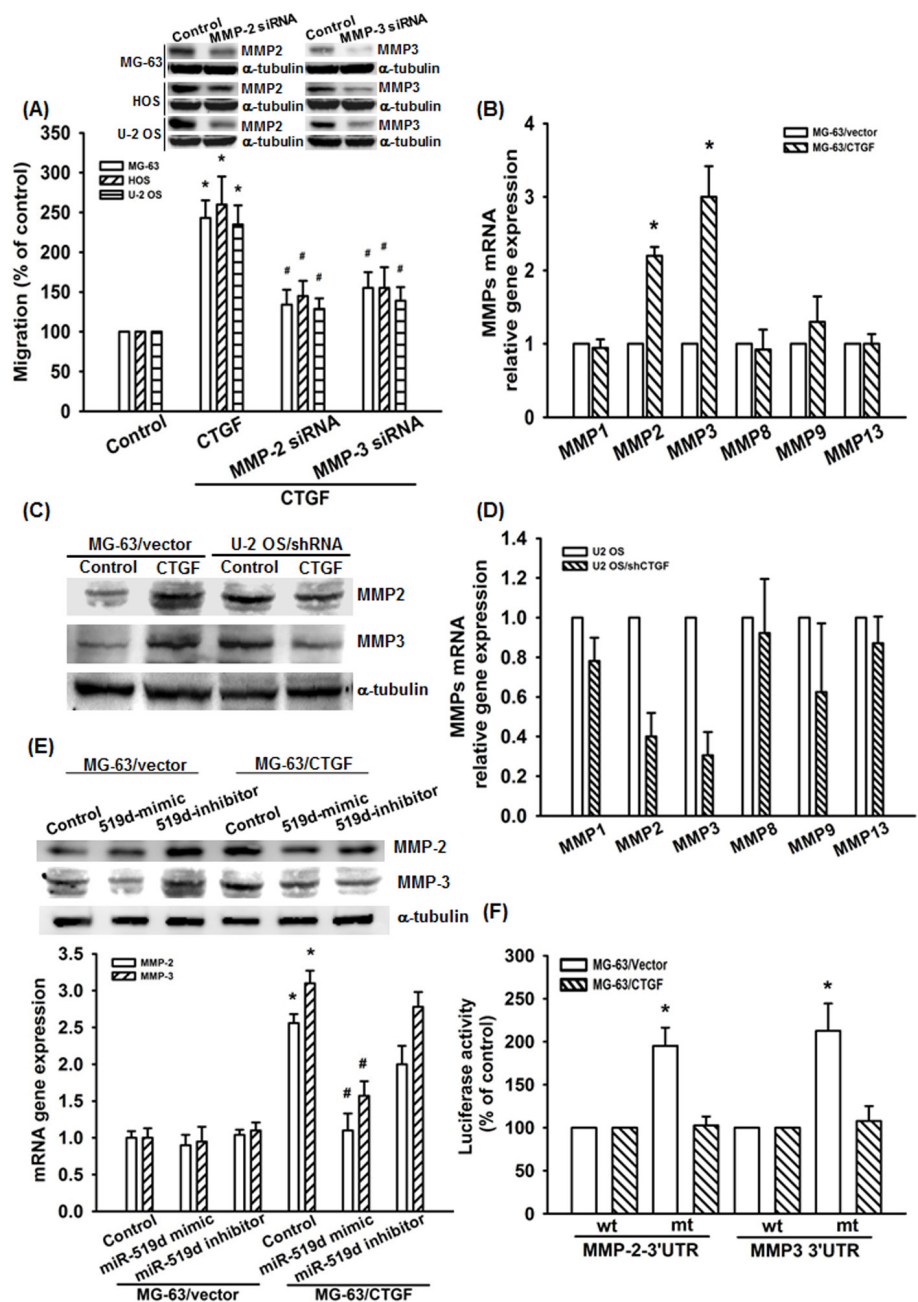
CTGF increases MMP-2 and MMP-3 expression and cell migration by down-regulating miR-519d (A) Cells were transfected with MMP-2 or MMP-3 siRNA for 24 h. The MMP2 and MMP-3 expression and cell migration were examined by western blot and Transwell assay. (B-D) The mRNA and protein expression in indicated cells was examined by q-PCR and western blot. (E) Cells were transfected with miR-519d mimic or inhibitor for 24 h and MMPs expression was examined by western blot and q-PCR. (F) Cells were transfected with indicated luciferase plasmid for 24 h, and the relative luciferase activity was measured. Results are expressed as mean ± SEM. *, p < 0.05 as compared with control group, and ^#^, p < 0.05 as compared with CTGF-treated control group.

To identify the mechanism of miR-519d-involved osteosarcoma migration, we searched for possible downstream genes using bioinformative screening analysis of miRNA target databanks: DIANA-mT, miRanda, miRDB, and Targetscan. From these four databanks overlapping between the predicted targets of miR-519d, the MMP-2 and MMP-3 were ranked as the most probable targets. We therefore hypothesized that both MMP-2 and MMP-3 were involved in CTGF-meidated migration. Transfection of cells with the MMP-2 or MMP-3 siRNA abolished CTGF-induced cell migration (Fig. 3A). Previous studies showed significant expression of MMP-1, -2, -3, -8, -9, and -13 in human osteosarcoma cells, and also indicating that these MMPs participated the progression and metastases in human osteosarcoma [31-34]. By using q-PCR and western blot, we found the mRNA and protein expression of MMP-2 and MMP-3 but not others were increased in MG-63/CTGF cells (Fig. 3B&C). In contrast, knockdown CTGF decreased MMP-2 and MMP-3 expression in U-2 OS/CTGF-shRNA cells (Fig. 3C&D). Furthermore, transfection with miR-519d mimic abolished CTGF-induced MMP-2 and MMP-3 expression (Fig. 3E). To examine whether miR-519d regulates the 3'UTR of MMP-2 and MMP-3, we constructed luciferase reporter vectors harboring the wild-type 3'UTR of the MMP-2 and MMP-3 mRNA (wt-MMP-2-3'UTR and wt-MMP-3-3'UTR) and vector containing mismatches in the predicted miR-519d binding site (mt-MMP-2-3'UTR and mt-MMP-3-3'UTR; Supplementary Fig. S1) and transfected these vectors into MG-63/CTGF and control cells. The results shown that CTGF increased luciferase activity in the wt-MMP-2-3'UTR and wt-MMP-3-3'UTR plasmid but not in the mt-MMP-2-3'UTR and mt-MMP-3-3'UTR (Fig. 3F). Taken together, these data demonstrated that miR-519d directly represses the MMP-2 and MMP-3 protein expression through binding to the 3'UTR of the human *MMP-2* and *MMP-3* gene.

### MEK/ERK pathway is involved in CTGF-mediated migration and suppression of miRNA-519d

**Figure 4:**
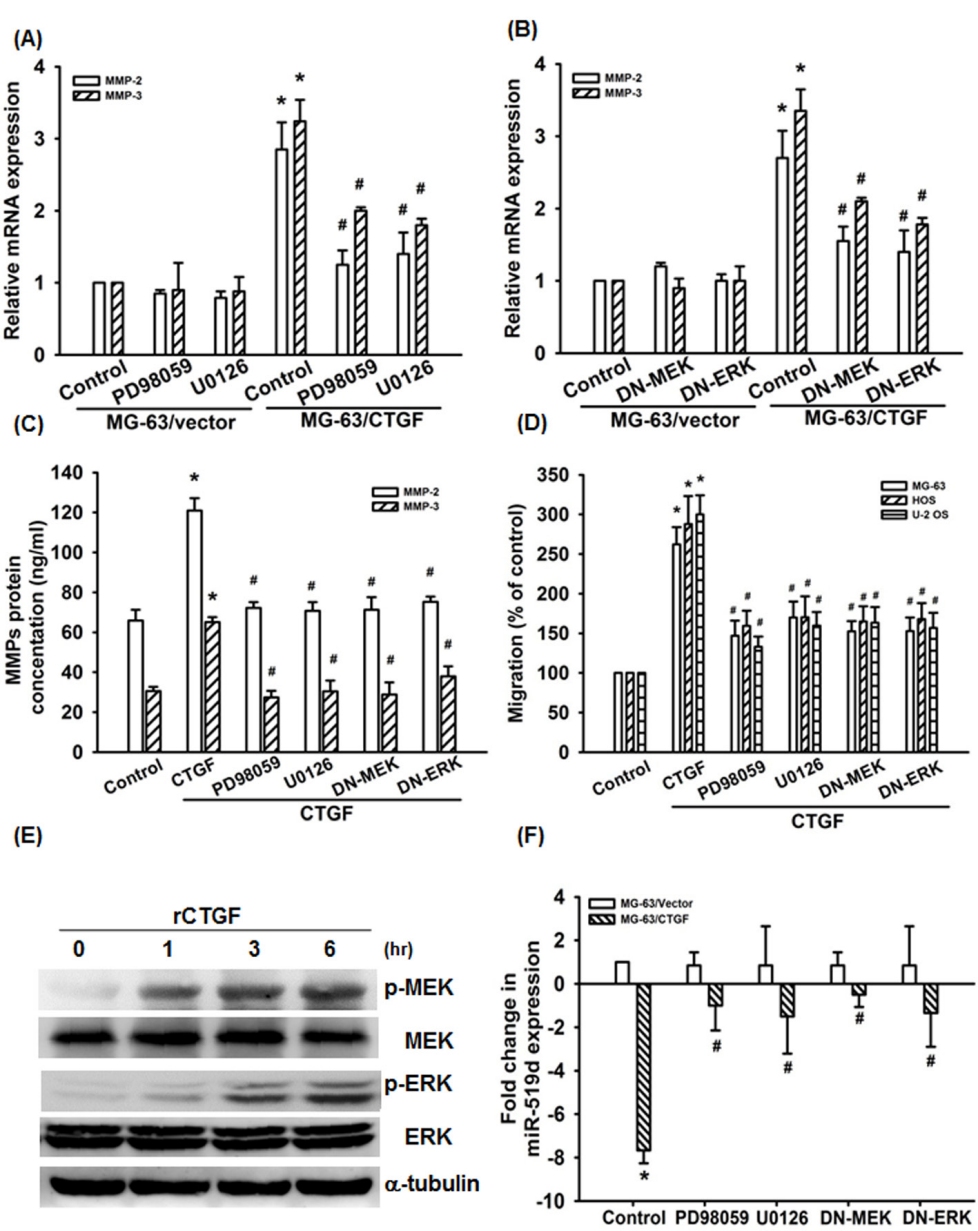
MEK/ERK pathway is involved in CTGF-induce migration and suppression of miRNA-519d (A-D) Cells were pretreated with PD98059 (10 μM) and U0126 (10 μM) for 30 min or transfected with MEK1 and ERK2 mutant for 24 h, the MMPs expression and cell migration was examined by q-PCR, ELISA, and Transwell assay. (E) MG-63 cells were incubated with rCTGF (50 ng/ml) for indicated time intervals, MEK and ERK phosphorylation examined by western blot. (F) Cells were pretreated with PD98059 (10 μM) and U0126 (10 μM) for 30 min or transfected with MEK1 and ERK2 mutant for 24 h followed, the miR-519d expression was examined by qRT-PCR. Results expressed as mean ± SEM. *, p < 0.05 as compared with controls, and ^#^, p < 0.05 as compared with CTGF-treated control gruop.

The MEK/ERK pathway has been reported to regulate MMPs expression and cancer metastasis [35-37]. We therefore examined whether the MEK/ERK is involved in CTGF-mediated cell migration and MMPs expression in osteosarcoma cells. Pretreatment of MG-63/CTGF cells with MEK inhibitors (PD98059 and U0126) or transfection with MEK1 and ERK2 mutant abolished CTGF-induced MMPs expression and cell migration (Fig. 4A-D). In addition, stimulation of cells with CTGF also promoted MEK and ERK phosphorylation time-dependently (Fig. 4E). Furthermore, MEK or ERK inhibitors and mutants reversed CTGF-inhibited miR-519d expression (Fig. 4F), indicating that CTGF increases MMPs and migration as well as suppresses miR-519d through MEK/ERK pathway.

### Knockdown of CTGF suppress lung metastasis *in vivo*

**Figure 5:**
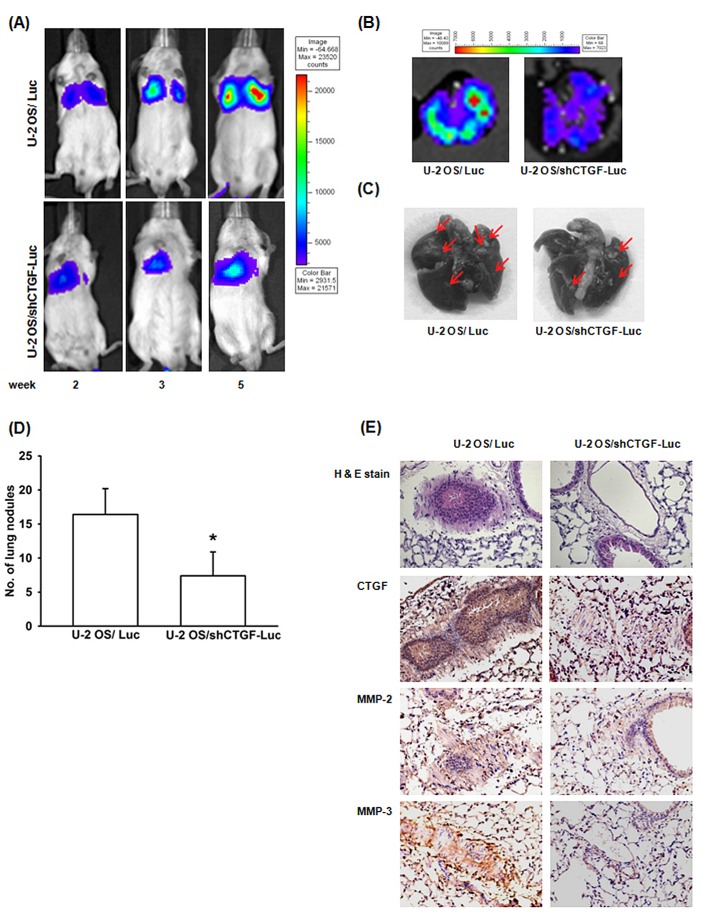
Knockdown of CTGF expression suppress lung metastasis *in vivo* (A) U-2 OS/Luc or U-2 OS/shCTGF-Luc cells were injected into the lateral tail vein, and lung metastasis was monitored by bioluminescence imaging at indicated time intervals. (B-E) After 6 weeks mice were sacrificed, their lungs were excised and photographed, tumor colonies counted and stained with CTGF, MMP-2, and MMP-3. Results are expressed as mean ± SEM. *, p < 0.05 as compared with U-2 OS/Luc group.

To determine whether knockdown of CTGF suppress lung metastasis *in vivo*, we established luciferase-expressing U-2 OS/Luc and U-2 OS/shCTGF-Luc cells. Cells were intravenously injected into SCID mice, and tumor metastasis was monitored by bioluminescence imaging. As shown in Fig. 5A, knockdown of CTGF significantly suppressed lung metastasis in the time course. Mice were sacrificed after 6 weeks injected, *ex vivo* imaging of the lungs derived from mice on day 42 showed a higher intensity and metastatic nodules in the U-2 OS/Luc versus U-2 OS/shCTGF-Luc group (Fig. 5B-D). Immunohistochemical staining also found expression of CTGF, MMP-2, and MMP-3 starkly decreasing in U-2 OS/shCTGF-Luc group (Fig. 5E). These indicates CTGF knockdown suppressing lung metastasis *in vivo*.

### Confirming significance of CTGF, MMPs, and miR-519d in clinical samples

**Figure 6:**
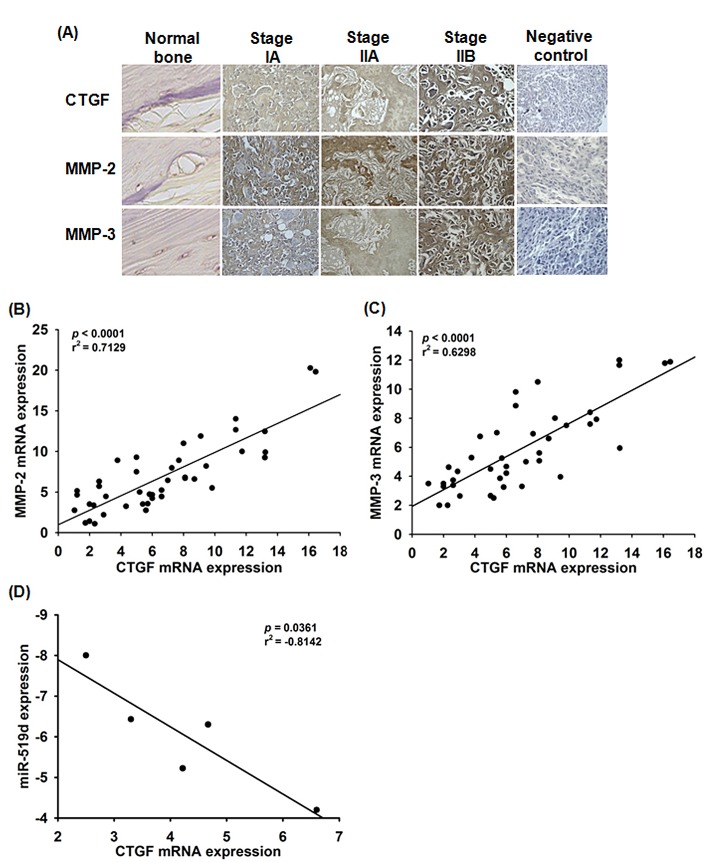
Clinical importance of CTGF, MMPs and miR-519d in osteosarcoma (A) IHC stain of CTGF, MMP-2, and MMP-3 in normal versus osteosarcoma tissue. Correlation between CTGF/MMP-2 (B), CTGF/MMP-3 (C) and CTGF/mi-519d (D) in human osteosarcoma.

Finally, we investigated clinical importance of CTGF, MMPs, and miR-519d in osteosarcoma patients. Expression of CTGF, MMP-2, and MMP-3 in osteosarcoma patients was significantly higher than in normal bone (Fig. 6A). Quantitative RT-PCR analysis of CTGF, MMP2, MMP-3, and miR-519d performed in osteosarcoma and normal samples, showed negative correlation between CTGF and miR-519d; positive correlation between CTGF and MMP-2, CTGF and MMP-3 (Fig. 6B-D). High CTGF expression was significantly associated with clinical stage and metastasis (Table 1); higher CTGF and lower miR-519d expression were linked with MMP-2 and MMP-3 expression, correlating with osteosarcoma development and metastasis.

**Table 1:** Correlation of CTGF expression with clinical status in osteosarcoma

Characteristic	CTGF low (n=21)	CTGF high (n=29)	p-value
Age (year)			
< 24	7	12	0.5629
> 25	14	17	
Gender			
Female	8	9	0.7635
Male	13	20	
Stage			
IA	4	2	0.0453*
IIA	9	7	
IIB	8	13	
IVB	0	7	
Tumor status			
T1	13	8	0.0152*
T2-T3	8	21	
Distant metastasis			
M0	21	22	0.0152*
M1	0	7	

* ,p <0.05

## DISCUSSION

Cancer is one of the leading causes of death worldwide [38]. Osteosarcoma is the most common primary bone tumor and with high morbidity that mainly occurs in children and adolescents. From a clinical perspective, primary osteosarcoma management can be successfully in most patients. But, the development of metastasis to the lung or other organs represents the most common cause of death [3]. Clear understanding of metastasis biology in osteosarcoma is necessary. This study characterized effect of CTGF on production of MMP-2 and MMP-3 in human osteosarcoma, which is responsible for subsequent increased migration and metastasis. Results indicated CTGF inducing upregulation of MMP-2 and MMP-3 expression in human osteosarcoma cells by down-regulating miR-519d through MEK and ERK pathway and contributing the tumor metastasis.

CTGF belongs to a family of secreted proteins that have a number of cellular functions, as well as pathologic processes [11]. In tumor metastasis, the double-sided sword effect of CTGF has been widely reported: CTGF promotes cancer growth or metastasis in chondrosarcoma, breast, and brain caners [10, 39]; in contrast, it inhibits tumor metastasis in oral and lung cancers [41, 42]. However, the role of CTGF in osteosarcoma progression and metastasis remain unknown. Our *in vitro* data shown that overexpression of CTGF in osteosarcoma cells enhanced migration ability. On the other hand, knockdown of CTGF expression *via* CTGF-shRNA strongly decreased the cell migration. By using the IHC staining, we also found that the expression of CTGF in osteosarcoma tissues were correlates with tumor stage and metastasis. Finally, we used the animal model to confirm that down-regulation of CTGF decreased lung metastasis *in vivo*. To the best of our knowledge, this is the first study to demonstrate that CTGF promotes osteosarcoma metastasis *in vitro* and *in vivo*.

Newly identified small noncoding miRNAs, belong to a novel class that control gene expression by binding to complementary sequences in 3'UTRs of target mRNAs [43, 44]. Deregulated miRNA expression has been cited in human cancer and may affect multiple steps during metastasis [45]. It has been revealed that miR-21 [46], 125b [47], 143 [48], 20a [49], and 93 [50] regulates metastatic ability in osteosarcoma. Our study screened 576 miRNAs from miRNome microRNA Profilers QuantiMir™ kit and found miR-519d most down-regulated in response to CTGF overexpression; miR-519d reportedly suppresses tumor formation in human hepatocellular carcinoma, making it an attractive candidate biomarker for prediction of anticancer treatment response [51, 52]. In this study, transfection of cells with miR-519d mimic reduced CTGF-induced cell migration, meaning miR-519d can be an anti-metastatic gene.

Previous study demonstrated that MMPs functioning in many stages of cancer metastasis, including enhanced escape of survival in circulation, and extravasation at the secondary site [53]. CTGF has also been reported to regulate MMP-2 and MMP-3 expression [54, 55]. Our study found mRNA and protein expression of MMP-2 and MMP-3 increasing in MG-63/CTGF cells. Knockdown of MMP-2 or MMP-3 expression via transfection with MMP-2 or MMP-3 siRNA abolished CTGF-induced osteosarcoma cell migration activity, meaning both MMP-2 and MMP-3 involved in CTGF-mediated cell migration. By using miRNA target prediction (DIANA-mT, miRanda, miRDB, and Targetscan), we proved MMP-2 and MMP-3 most probable miR-519d targets; transfection of miR-519d-mimic strongly inhibited CTGF-induced MMP-2 and MMP-3 expression. We indicated miR-519d directly repressing MMP-2 and MMP-3 protein expression through binding to 3'UTR of human *MMP-2* and *MMP-3* genes, thus negatively regulating MMP-2 and MMP-3-mediated metastasis.

Metastasis is caused by cells wandering from a primary tumor, then colonizing other sites in the body. Most common features are described as invasion, intravasation or extravasation from the circulatory system, colonization and angiogenesis at distant sites. The latter is generally connected with poor prognosis of osteosarcoma patients. Development of anti-metastatic therapy could conceivably be useful in such cases. Our results point to high CTGF expression as significantly associated with clinical stage and metastasis. CTGF increases MMP-2/-3 expression and promotes metastasis by down-regulating miR-519d through MEK and ERK pathways; CTGF inhibition presents a new therapeutic target.

## MATERIALS AND METHODS

### Materials

Anti-rabbit and anti-mouse IgG-conjugated horseradish peroxidase, rabbit polyclonal antibody specific for α-tubulin, and mouse monoclonal antibodies specific for CTGF, MMP-2, and MMP-3 were purchased from Biotechnology (Santa Cruz, CA). ON-TARGETplus siRNA of MMP2, MMP3, and control were purchased from Dharmacon Research (Lafayette, CO). PD98059, U0126 and human MMP-2 and MMP-3 enzyme-linked immunosorbent assay (ELISA) kits were purchased from Calbiochem (San Diego, CA). Recombinant human CTGF was purchased from PeproTech (Rocky Hill, NJ). MiRNome microRNA Profilers QuantiMir™ kit was from System Biosciences (SBI) (Mountain View, CA). MiR-519d mimic and inhibitor were purchased from Invitrogen (Carlsbad, CA). MEK1 dominant-negative mutant was a gift from Dr. W. M. Fu (National Taiwan University at Taipei). ERK2 dominant-negative mutant was a gift from Dr. M. Cobb (Southwestern Medical Center, Dallas, TX), all other chemicals purchased from Sigma-Aldrich (St. Louis, MO).

### Cell culture

The human osteosarcoma cell lines (MG-63, U-2 OS, and HOS) were purchased from the American Type Cell Culture Collection (Manassas, VA, USA). MG-63 and HOS cells were maintained in Dulbecco's Modified Eagle Medium (DMEM) and U-2 OS cells were maintained in McCoy's 5A medium, which was supplemented with 20 mM HEPES and 10% heat-inactivated FCS, 2 mM glutamine, penicillin (100 U/ml), streptomycin (100 μg/ml) and 10% FBS at 37 °C with 5% CO_2_.

### Patients and specimen preparation

The study protocol was approved by the Institutional Review Board of China Medical University Hospital, and all subjects gave informed written consent before enrollment. The specimens of tumor tissue or normal bone were obtained from patients who were had been diagnosed with osteosarcoma and had undergone surgical resection at China Medical University Hospital.

### Migration assay

The migration assay was performed using Transwell (Costar, NY; pore size, 8 mm). Approximately 1×10^4^ cells in 100 ml of serum-free medium were placed in the upper chamber, and 300 ml of the same medium was placed in the lower chamber. The plates were incubated for 24 h at 37°C in 5% CO_2_, cells were then fixed in 3.7% formaldehyde for 15 min and stained with 0.05% crystal violet in PBS for 15 min. Cells on the upper side of the filters were removed with cotton-tipped swabs, and the filters were washed with PBS. Cells on the underside of the filters were examined and counted under a microscope. Each clone was plated in triplicate for each experiment, and each experiment was repeated at least three times.

### Immunohistochemical (IHC) staining

Human osteosarcoma tissue sections were deparaffinized with xylene and rehydrated through addition of ethanol. Endogenous peroxidase activity was blocked with 3% hydrogen peroxide in methanol for 10 min. Heat-induced antigen retrieval was carried out for all sections in 0.01 M sodium citrate buffer, pH 6 at 95°C for 25 min. Human CTGF, MMP-2, and MMP-3 antibodies were applied at a dilution of 1:200 and incubated at 4°C overnight. The antibody-binding signal was detected using the NovoLink Polymer Detection System (Leica Microsystems) and visualized with the diaminobenzidine reaction. The sections were counterstained with hematoxylin. The immunohistochemistry results were scored by taking into account the percentage of positive detection and intensity of the staining.

### ELISA assay

Human osteosarcoma cells were cultured in 24-well plates. After reaching confluence, cells were changed to serum-free medium. Cells were then treated with CTGF alone for 24 h, or pretreated with pharmacological inhibitors or transfected with specific siRNA followed by stimulation with CTGF for 24 h. After treatment, the medium was removed and stored at -80°C. Then, MMP-2 and MMP-3 in the medium was determined using MMP-2 and MMP-3 ELISA kit according to the manufacturer's protocol.

### Luciferase activity assay

3' UTR regions of human MMP-2 and MMP-3 genes were amplified by PCR. The 3' UTR were cloned in pGL2-Control vector (Promega, Madison, WI, USA) downstream of the reporter gene. The predicted MMP-2 and MMP-3 binding sites, identified by the miRDB (http://mirdb.org/miRDB), were amplified by PCR and cloned in pGL2-Control vector upstream of the reporter gene (Supplementary Fig. S1). Mutant plasmids were generated using a QuickChange Site-Directed Mutagenesis kit (Stratagene, Cedar Creek, TX, USA).

### Western blot analysis

Protein concentration was determined using the Thermo Scientific Pierce BCA Protein Assay Kit (Thermo Fisher Scientific Inc., USA). Proteins were resolved on SDS-PAGE and transferred to immobilon polyvinyl difluoride (PVDF) membranes. The blots were blocked with 4% BSA for 1 h at room temperature and incubated with the following primary antibodies for 1 h at room temperature. After 3 washes in tris-buffered saline with 0.05% Tween 20 (TBS-Tween), the blots were subsequently incubated with a donkey anti-rabbit or anti-mouse peroxidase-conjugated secondary antibody for 1 h at room temperature. The blots were visualized by enhanced chemiluminescence using Kodak X-OMAT LS film (Eastman Kodak, Rochester, NY). Quantitative data were obtained using a computing densitometer and ImageQuant software (Molecular Dynamics, Sunnyvale, CA).

### Quantitative real-time PCR

Total RNA was extracted from osteosarcoma cells using a TRIzol kit (MDBio Inc., Taipei, Taiwan). The reverse transcription reaction was performed using 2 μg of total RNA that was reverse transcribed into cDNA using an oligo(dT) primer. Quantitative real-time polymerase chain reaction (q-PCR) analysis was carried out using TaqMan^®^ one-step PCR Master Mix (Applied Biosystems, Foster City, CA, USA). Total complementary DNA (100ng/25μL reaction) was mixed with sequence-specific primers and TaqMan^®^ probes according to the manufacturer's instructions. Sequences for all target gene primers and probes were purchased commercially (β-actin was used as the internal control) (Applied Biosystems). Q-PCR assays were carried out in triplicate using a StepOnePlus sequence detection system. The cycling conditions were 10 min of polymerase activation at 95 °C, followed by 40 cycles at 95 °C for 15 s and 60 °C for 60 s.

For miRNAs detection, reverse transcription was performed using Mir-X™ miRNA First-Strand Synthesis and SYBR^®^ qRT-PCR (Clontech Laboratories, Inc., CA, USA). U6 snRNA levels were used for normalization. The specific forward primer of miR-519d was as follows: 5'-CAAAGTGCCTCCCTTTAGAGTG-3'. Forward and reverse primers for U6 were 5'-CTCGCTTCGGCAGCACATATACT A-3'and 5'-ACGAATTTGCGTGTCATCCTTGCG-3'. The threshold was set above the non-template control background and within the linear phase of target gene amplification to calculate the cycle number at which the transcript was detected (denoted as CT).

### *In vivo* metastasis model

Cells (U-2 OS/Luc or U-2 OS/shCTGF-Luc) were washed and resuspended in PBS. Subsequently, a unit suspension containing 5×10^6^ cells in 100 μl PBS was injected into the lateral tail vein of 5-week-old severe combined immune deficient (SCID) mice. Lung metastasis was monitored using an *in vivo* imaging system (Xenogen IVIS imaging system). After 6 weeks, the mice were sacrificed by overdose with anesthetic. The lungs were removed, photographed, and counted tumor colonies. The lungs were then fixed in 10% formalin, embedded in paraffin and subsequently processed for IHC staining with CTGF, MMP-2 and MMP-3.

### Statistics

Data are expressed as the mean ± standard error. The differences between groups were analyzed using the Student's *t*-test or the χ^2^ test of variance. The difference was considered significant if the *p* value was less than 0.05.

## SUPPLEMENTARY MATERIAL


