# Correction: CTGF increases matrix metalloproteinases expression and subsequently promotes tumor metastasis in human osteosarcoma through down-regulating miR-519d

**DOI:** 10.18632/oncotarget.27459

**Published:** 2020-01-28

**Authors:** Hsiao-Chi Tsai, Hong-Lin Su, Chun-Yin Huang, Yi-Chin Fong, Chin-Jung Hsu, Chih-Hsin Tang

**Affiliations:** ^1^ Department of Life Sciences, National Chung Hsing University, Taichung, Taiwan; ^2^ Department of Orthopaedic Surgery, China Medical University Beigang Hospital, Yun-Lin County, Taiwan; ^3^ Graduate Institute of Clinical Medical Science, China Medical University, Taichung, Taiwan; ^4^ School of Chinese Medicine, College of Chinese Medicine, China Medical University, Taichung, Taiwan; ^5^ Department of Orthopedic Surgery, China Medical University Hospital, Taichung, Taiwan; ^6^ Graduate Institute of Basic Medical Science, China Medical University, Taichung, Taiwan; ^7^ Department of Pharmacology, School of Medicine, China Medical University, Taichung, Taiwan; ^8^ Department of Biotechnology, College of Health Science, Asia University, Taichung, Taiwan

**This article has been corrected:** Due to errors during figure preparation, the images for Figure 3C’s MMP-2 blot and Figure 3E’s MMP-3 blot are presented as duplicates. The corrected Figure 3C image is shown below. The authors declare that these corrections do not change the results or conclusions of this paper.

Original article: Oncotarget. 2014; 5:3800–3812. 3800-3812. https://doi.org/10.18632/oncotarget.1998

**Figure 3:**
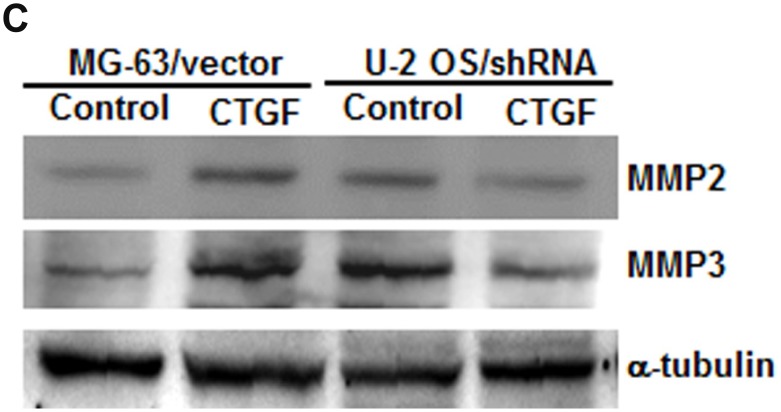
CTGF increases MMP-2 and MMP-3 expression and cell migration by down-regulating miR-519d (**A**) Cells were transfected with MMP-2 or MMP-3 siRNA for 24 h. The MMP2 and MMP-3 expression and cell migration were examined by western blot and Transwell assay. (**B**–**D**) The mRNA and protein expression in indicated cells was examined by q-PCR and western blot.

