# Supplementary material for: CTGF increases matrix metalloproteinases expression and subsequently promotes tumor metastasis in human osteosarcoma through down-regulating miR-519d
Source: Oncotarget. 2014 May 21;5(11):3800–12. doi: 10.18632/oncotarget.1998 (PMC4116521; doi:10.18632/oncotarget.1998)
Supplement: Supplementary file 1 [file oncotarget-05-3800-s001.pdf]

CTGF increases matrix metalloproteinases expression and subsequently promotes tumor metastasis in human osteosarcoma through down-regulating miR-519d

Supplementary Material

Table S1: Representative miRNA expression after overexpressed CTGF. qRT-PCR analysis of > 3-fold expression of miRNAs in MG-63/vector and MG-63/CTGF cells.

Up-regulated Target ID	Fold	Down-regulated Target ID	Fold
hsa-miR-202	10.98	hsa-miR-519d	0.000000014
hsa-miR-541	10.18	hsa-miR-483-5p	0.00000017
hsa-miR-483-3p	9.39	hsa-miR-222	0.0000027
hsa-miR-34c-3p	8.14	hsa-miR-93	0.0000027
hsa-miR-370	6.51	hsa-miR-130a	0.000029
hsa-miR-92b	6.46	hsa-miR-23a	0.000031
hsa-miR-126	6.07	hsa-miR-23b	0.00032
hsa-miR-539	5.01	hsa-miR-92a	0.00033
hsa-miR-323-5p	3.47		
hsa-miR-382	3.09		

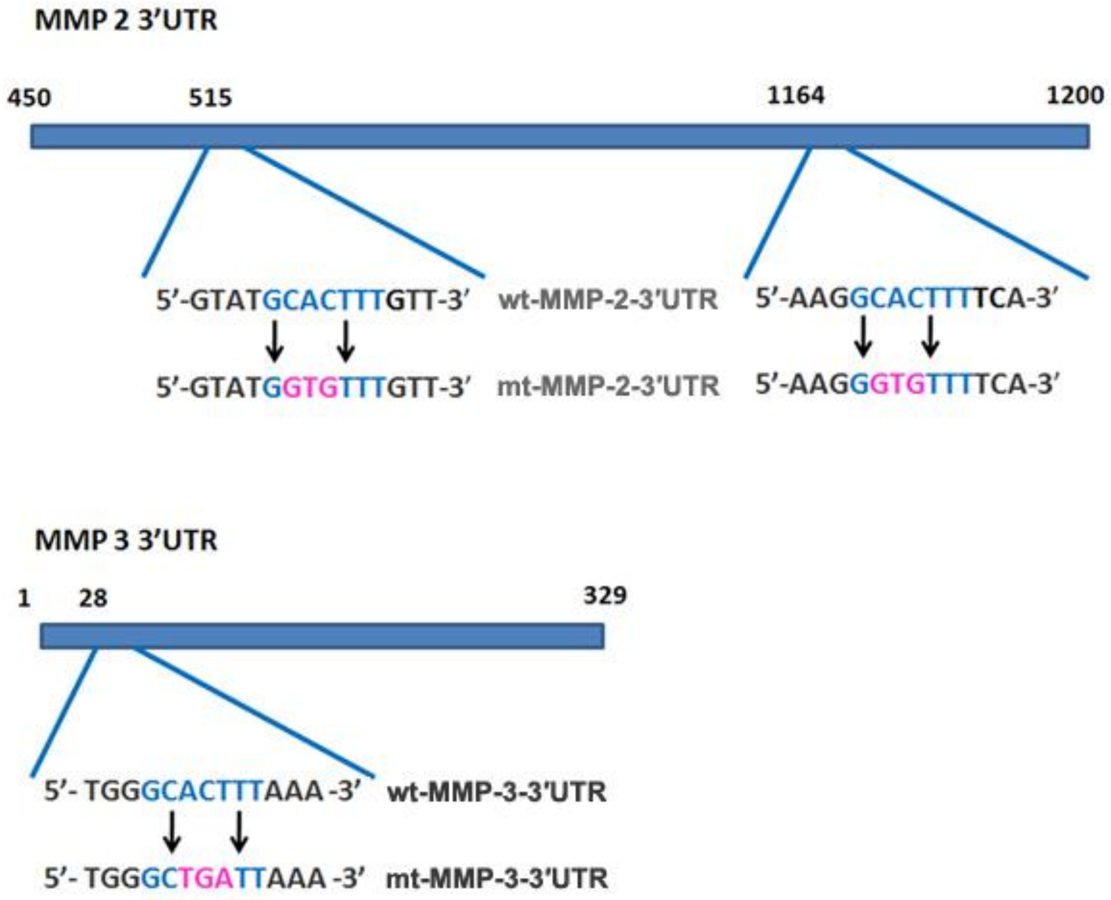

Figure S1: Schematic illustrations of the pGL2 luciferase reporter construct for examining the effect of miR-519d on MMP-2 and MMP-3 3'UTR. The full-length sequence of the MMP-2 and MMP-3 3'UTR is located at position 2295 and 1500 of MMP-2 mRNA (NM_004530) and MMP-3 mRNA (NM_002422). The miR-519d seed location in MMP-2 3'UTR are 515 and 1164, and in MMP-3 3'UTR is 28. The fragment that contains the predicted miR-519d binding site was cloned into luciferase report plasmids. The construct with the MMP-2 and MMP-3 3'UTR mutated at the predicted miR-519d binding site are also depicted.

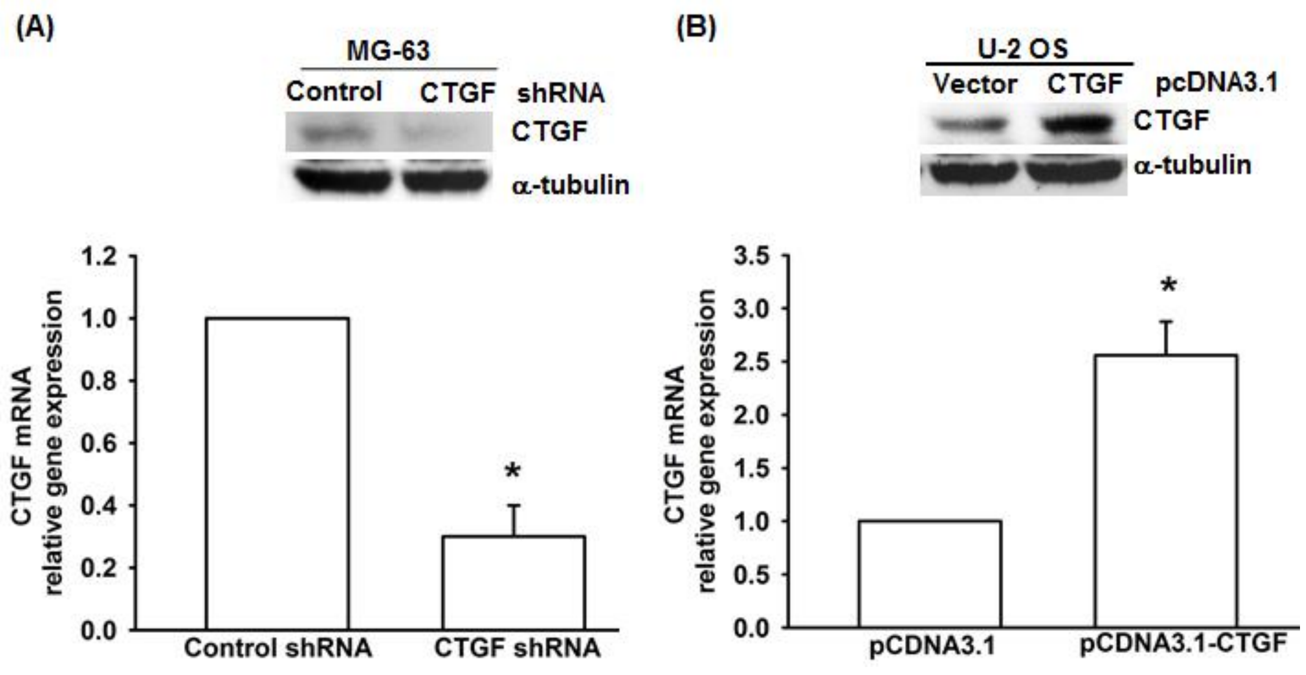

Figure S2: The CTGF expression in osteosarcoma cells. MG-63 cells were transfected with control or CTGF shRNA (A) and U-2 OS cells were transfected with pCDNA3.1 or CTGF-overexpressing pCDNA3.1 vector (B). The protein and mRNA expression of CTGF were examined by western blot and q-PCR. *, $p < 0.05$ as compared with control group.
